# *Trichinella* detection in wild boar meat intended for private consumption: Insights from a European study

**DOI:** 10.1016/j.fawpar.2026.e00358

**Published:** 2026-09-03

**Authors:** Mariana Rodrigues, Edoardo Pozio, Vincenzo Veneziano, Famke Jansen, Thomai Lazou, Sorin Daniel Dan, Miroslaw Różycki, Jelena Petrović, Madalena Vieira-Pinto

**Affiliations:** a Department of Veterinary Sciences, University of Trás-os-Montes and Alto Douro, 5000-801 Vila Real, Portugal; b Vasco da Gama Research Centre, Vasco da Gama University School, Av. José R Sousa Fernandes, 297, 3020-210 Coimbra, Portugal; c Animal and Veterinary Research Center (CECAV), University of Trás-os-Montes and Alto Douro, 5000-801 Vila Real, Portugal; d Former Department of Infectious, Parasitic and Immunomediated Diseases, Italian National Institute of Health, Viale Regina Elena 299, 00161 Rome, Italy; e Department of Veterinary Medicine and Animal Production, University of Naples Federico II, Via F. Delpino, 1, 80137 Naples, Italy; f Institute of Tropical Medicine (ITM), Department of Biomedical Sciences, 155 Nationalestraat, B-2000 Antwerp, Belgium; g Laboratory of Animal Food Products Hygiene - Veterinary Public Health, School of Veterinary Medicine, Aristotle University of Thessaloniki, GR 54124 Thessaloniki, Greece; h Department of Animal Production and Food Safety, University of Agricultural Sciences and Veterinary Medicine, Faculty of Veterinary Medicine, 3-5 Mănăştur Street, 4003720 Cluj Napoca, Romania; i Department of Preclinical Sciences and Infectious Diseases, Poznań University of Life Science, Poznan 60-637, Poland; j Department of Epizootiology, Clinical Examination and DDD, Scientific Veterinary Institute “Novi Sad”, Novi Sad, Serbia

**Keywords:** Europe, Private consumption, *Trichinella*, Wild boar, Zoonosis, Hunting, Game meat

## Abstract

Private consumption of wild boar meat is increasingly widespread across Europe. Notably, only a small proportion of hunted game is processed through approved establishments, while the majority is intended for private consumption. Testing wild boar meat for *Trichinella* spp. is mandatory when the meat is placed on the market. However, for private consumption this testing may not be carried out, raising public health concerns due to the risk of *Trichinella* spp. infection. To assess this risk, a survey was conducted in 26 European countries to collect data on the number of hunted wild boars (*Sus scrofa*) intended for private consumption and the prevalence of *Trichinella* spp. in wild boars, and to compare legislation and operational practices related to testing requirements, sample handling, diagnostic methods, training requirements, reporting procedures, mandatory measures for positive samples, penalties for non-compliance, and awareness-raising activities. Findings revealed significant heterogeneity in testing obligations, sample handling procedures, laboratory requirements, and enforcement mechanisms. While some countries mandate systematic testing and subsidize costs, others rely on voluntary testing or impose no requirements, leaving consumers at higher risk. Reported prevalence of *Trichinella* spp. in wild boar varied widely, ranging from 0% to over 6% across countries and years, though data availability was often inconsistent. Awareness campaigns and hunter training remain limited in many regions, contributing to underreporting, low compliance, and insufficient risk perception. Key challenges include lack of hunter engagement, insufficient testing facilities, and variable penalties for non-compliance. To mitigate risks, we recommend harmonizing surveillance strategies, expanding education and communication initiatives, ensuring accessible and affordable testing, and strengthening collaboration between veterinary authorities and hunting communities. Such measures are essential to reduce the zoonotic risk of trichinellosis linked to privately consumed wild boar meat in Europe.

## 1 Introduction

Trichinellosis in humans is a serious and potentially fatal food-borne zoonosis with worldwide distribution, caused by the larval stage of tissue nematodes of the genus *Trichinella*. Transmission of *Trichinella* spp. to various hosts, including humans, occurs through the ingestion of raw or undercooked meat from infected animals (Gottstein et al., 2009).

Europe faces challenges in the prevention and control of trichinellosis due to the presence of *Trichinella* spp., which is common in wild susceptible animals (van der Giessen et al., 2021). The European Commission (EC) has designated nematodes of the genus *Trichinella* among the zoonotic agents that are subject to mandatory surveillance within the Union (Anonymous., 2003). Subsequently, the EC adopted the Commission Implementing Regulation (EU) 2015/1375 (European Commission (EC), 2015), which establishes specific provisions governing official controls for *Trichinella* spp. in meat. All meat-producing animals susceptible to *Trichinella* infection, such as domestic pigs, wild boars, and bears, must undergo post-mortem examination if their meat is intended for placement on the market. Placing on the market refers to the holding of food or feed for the purpose of sale, including offering for sale or any other form of transfer, whether free of charge or not. Conversely, carcasses of animals intended for private consumption may be exempted from official controls. Nonetheless, private consumption of game meat warrants particular attention, as only a limited proportion of hunted game is processed in approved game-handling establishments (EFSA BIOHAZ Panel, 2013). Since the second half of the 20th century, the wild boar (*Sus scrofa*) population in Europe has been steadily increasing, leading to the increased consumption of wild boar meat and amplifying public health concerns (Abrantes and Vieira-Pinto, 2023).

Since 2015, the prevalence of human trichinellosis in Europe has remained extremely low. Annual European Centre for Disease and Control (ECDC) and European Food Safety Authority (EFSA) reports describe low numbers of infections each year, usually a few dozen to a few hundred across all EU/EEA countries (EFSA and ECDC (European Food Safety Authority and European Centre for Disease Prevention and Control), 2023, EFSA and ECDC (European Food Safety Authority and European Centre for Disease Prevention and Control), 2024, EFSA and ECDC (European Food Safety Authority and European Centre for Disease Prevention and Control), 2025), which is negligible when compared with the major foodborne zoonoses such as campylobacteriosis or salmonellosis. Over the period 2017–2021, trend analysis of trichinellosis by ECDC showed no significant overall increase or decrease in the EU notification rate, indicating a relatively stable but persistently low endemic level. In 2022–2023, less than one hundred human cases were reported in 28 EU/EEA countries, corresponding to an EU notification rate of 0.01–0.02 per 100,000 population (EFSA and ECDC (European Food Safety Authority and European Centre for Disease Prevention and Control), 2024).

In wild boar populations across Europe, *Trichinella* infection shows a parasitological prevalence (presence of larvae in muscles) ranging from 0.0% to 6.2% (EFSA and ECDC (European Food Safety Authority and European Centre for Disease Prevention and Control), 2023, EFSA and ECDC (European Food Safety Authority and European Centre for Disease Prevention and Control), 2024, EFSA and ECDC (European Food Safety Authority and European Centre for Disease Prevention and Control), 2025). Serological studies indicate higher figures, with seroprevalence of 2.2% in Italy, 6.4% in Greece and 17.4% in Estonia (Gómez-Morales et al., 2014; Karssin et al., 2016; Touloudi et al., 2015), but these data should be interpreted with caution since antibodies are detectable for years after the disappearance of the larvae from the muscles and due to the greater sensitivity of serological methods compared to parasitological ones (Bruschi et al., 2019; Pozio et al., 2020). In Europe, despite the relatively low infection rate of *Trichinella* spp. in wild boars, their meat represents one of the main sources of trichinellosis outbreaks in humans since most wild boar carcasses are not tested for *Trichinella* spp. (Diaz et al., 2020). Human trichinellosis is rare in Europe, but *Trichinella* spp. continues to circulate in both domestic and sylvatic cycles, with four species commonly reported: *T. spiralis, T. britovi, T. nativa, and T. pseudospiralis*. Species distribution is geographically heterogeneous, and composition differs among hosts and countries, reflecting local ecological and epidemiological conditions (Bilska-Zając et al., 2020; Iacob et al., 2022). In wild boars, *T. spiralis* and *T. britovi* are the species most often detected, although other species and mixed infections have also been reported (Balić et al., 2020). Importantly, these species differ markedly in their tolerance to low temperatures, and the widespread belief among hunters and consumers that freezing wild boar meat for a few weeks is sufficient to ensure its safety is misleading, as this practice may inactivate *T. spiralis* but not freezing-resistant species such as *T. britovi* or *T. nativa* (Pozio et al., 2006). Several human outbreaks in Europe have been linked to the consumption of raw or undercooked wild boar meat or homemade products, including cases in Spain, Germany, Italy, Belgium, France and Poland (Fichi et al., 2015; Gallardo et al., 2007; Peju et al., 2023; Różycki et al., 2022; Turiac et al., 2017). These events highlight the continuing public health relevance of wild boar meat, especially when carcasses are not tested and meat is consumed in private settings.

Given that wild boar is among the most extensively hunted game species in Europe, a European survey was conducted to assess the risks of trichinellosis. The aims of this study were to systematically collect data on the prevalence of *Trichinella* spp. in wild boar carcasses intended for private consumption, to compare applicable legal frameworks and operational practices, and to identify regulatory discrepancies, prevalence patterns, awareness initiatives, and principal risk factors in 26 European countries.

## 2 Material and methods

### 2.1 Study design

A cross-sectional survey was conducted within the framework of the COST Action *SafeGameMeat* (CA22166), coordinated by the University of Trás-os-Montes e Alto Douro (Vila Real, Portugal), involving members from 26 European countries. Each COST Action member identified a single knowledgeable entity in their respective country (i.e., veterinary authority, hunting association, reference laboratory or academic institution) with a recognized expertise in game meat safety.

### 2.2 Survey tool

A custom-designed structured questionnaire (Supplementary File 1) was developed and administered to the identified expert of each country to obtain country-specific data on *Trichinella* spp. in wild boar meat intended for private consumption. The draft questionnaire was subjected to a preliminary validation by seven representatives from different countries to assess its clarity, the suitability of wording, and the average time needed for completion. The questionnaire addressed multiple aspects of *Trichinella* spp. testing and control in wild boar meat intended for private consumption across European countries, including: i) legal and practical requirements for *Trichinella* spp. testing of wild boar meat intended for private consumption; ii) the flow of data from the field to government institutions including national and regional regulatory frameworks and enforcement mechanisms; iii) data collection on the numbers of wild boars intended for private consumption, and the prevalence of *Trichinella* spp. in tested animals; iv) awareness and communication initiatives conducted over the past two years (2023/2024) targeting hunters and the general population regarding *Trichinella* spp. control and prevention; v) mandatory testing policies for self-consumed wild boar meat, muscle type (i.e., diaphragm, masseter, intercostal, shoulder, foreleg, tongue, other), minimum quantity of muscle specimens to be sampled, and responsible parties for sample collection, submission and testing; vi) laboratory procedures encompassing sample handling, storage, and transport requirements, along with authorized diagnostic methods; vii) requirements for laboratory accreditation, personnel training protocols, financial support systems for testing, mandatory reporting obligations, applicable penalties in case of non-compliance, and actions taken upon positive detection; and viii) identification of main challenges in *Trichinella* spp. control and suggestions for practical improvements aimed at enhancing surveillance, compliance, and public health protection.

### 2.3 Data collection

The questionnaire was administered online between March and April 2025, targeting entities or individuals with expertise at the national level. Informed consent was obtained from all participants and data confidentiality was assured. The questionnaire included both closed and open-ended questions to collect quantitative data (e.g., numbers of wild boars, prevalence rates, costs) and qualitative information (e.g., challenges, awareness efforts, suggestions). In total, representatives from 26 European countries (Albania, Austria, Belgium, Bosnia and Herzegovina, Bulgaria, Croatia, Czech Republic, Finland, France, Germany, Greece, Italy, Latvia, Lithuania, Netherlands, North Macedonia, Norway, Poland, Portugal, Romania, Serbia, Slovakia, Spain, Sweden, Turkey and the United Kingdom) participated in the survey. The data flow from the field to the governmental institution was provided by six countries only (Table 2).

### 2.4 Data analysis

Data were analyzed descriptively and consolidated per country regarding prevalence practices and legislation. A comparative analysis was carried out to assess and compare regulatory frameworks and procedural practices across countries. To contextualize and compare the data collected on private consumption, published data from the EFSA and the ECDC regarding wild boar tested for *Trichinella* spp. were used, along with hunting activity statistics from the Federation of Associations for Hunting and Conservation of the European Union (EFSA and ECDC (European Food Safety Authority and European Centre for Disease Prevention and Control), 2023, EFSA and ECDC (European Food Safety Authority and European Centre for Disease Prevention and Control), 2024, EFSA and ECDC (European Food Safety Authority and European Centre for Disease Prevention and Control), 2025; FACE, 2025).

## 3 Results and discussion

### 3.1 Data on Trichinella spp. testing and prevalence in wild boar meat intended for private consumption

The number of wild boar carcasses intended for private consumption and the prevalence of *Trichinella* spp. infection in these animals were provided by eight countries (Austria, Belgium, Bulgaria, Croatia, North Macedonia, Poland, Romania, Slovakia) (Table 1). In Austria and Belgium, the number of wild boar carcasses intended for private consumption was obtained indirectly by subtracting the number of wild boars whose meat was intended for trade from the total number of hunted wild boars. In Bulgaria, the flow of information is managed directly by hunters through an app (Tanchev and Balieva, 2023); however, the proportion of hunters reporting data through the app is unknown. In Croatia and Poland, the spread of African Swine Fever (ASF) and the risk of transmission to domestic pigs have increased controls on hunted animals for both ASF and *Trichinella* spp. Comparison of data collected from the questionnaire (Table 1) with available literature data (FACE, 2025 and EFSA and ECDC (European Food Safety Authority and European Centre for Disease Prevention and Control), 2023, EFSA and ECDC (European Food Safety Authority and European Centre for Disease Prevention and Control), 2024, EFSA and ECDC (European Food Safety Authority and European Centre for Disease Prevention and Control), 2025) (Table 3) showed that the number of wild boars intended for private consumption in Romania was considerably higher. In this country, muscle samples from hunted wild boars are tested in private laboratories using trichinoscopy, a method much less sensitive than the recommended (European Commission (EC), 2015) artificial digestion; positive samples are subsequently tested in public laboratories using artificial digestion. This testing protocol suggests that the number of wild boars positive for *Trichinella* spp. is much higher than reported due to the low sensitivity of the test used. The flow of information from hunters to the final government institution responsible for collecting these data is summarized in Table 2.

**Table 1:** Number of hunted wild boar carcasses intended for private consumption, number of carcasses tested for *Trichinella* spp., and number of *Trichinella* positive carcasses by country from 2022 to 2024.

Country	Year^1^	No. of hunted wild boar intended for private consumption	No. of *Trichinella* spp. positive/tested wild boar intended for private consumption (%)	Mandatory test
Austria	2023	19,641	n.a.^2^	No

Belgium	2022	14,880	n.a.^2^	No

Bulgaria	2022	11,108	329/11,108 (2.96)	Yes
	2023	13,075	724/13,075 (5.53)	
	2024	15,305	920/15,305 (6.01)	

Croatia	2023	45,097	65/4888 (1.3)	Yes
	2024	41,603	64/3835 (1.7)	

North Macedonia	2023	n.a.^2^	14/4610 (0.3)	Yes
	2024	n.a.^2^	10/2445 (0.4)	

Poland	2022	162,344	220/162,344 (0.1)	Yes
	2023	177,399	155/177,399 (<0.1)	
	2024	187,516	169/187,516 (<0.1)	

Romania	2023	14,731	26/2692 (0.01)	Yes

Slovakia	2024	n.a.^2^	0/4827 (0)	No

1 Only years with available data are reported.

2 Data not available.

**Table 2:** Data flow from hunters to the final government institution.

Country	Primary notifier	Reporting pathway	Competent authority
Austria^1^	Unknown	District administrative authority → Province administrative authority	Federal Ministry of Agriculture and Forestry
Belgium^1^	Hunters	Game management authority	Regional competent authorities^2^
Bulgaria	Hunters	Registration via the “Hunt app” of hunted wild boar and samples for *Trichinella*/ASF test results	Bulgarian Food Safety Agency
Croatia	Hunter leader Hunters	Expert responsible for the implementation of the hunting economic basis → Hunting management database	Hunting Directorate, Ministry of Agriculture
Poland	Hunters	Regional Veterinary Hygiene Laboratories	Polish General Veterinary Inspectorate
Romania	Hunters	Private veterinarian → County Veterinary and Food Safety Authority Directorate → Game management authority	National Veterinary Health and Food Safety Authority

1 The number of wild boars intended for private consumption is deducted by subtracting the number of wild boars sent to game handling establishments from the total number of wild boars hunted.

2 Flanders: The agency for nature and forests; Wallonia: Department of the study of the natural and agricultural environment; Brussels: Living environment Brussels.

#### 3.1.1 Data collection

Across the six countries that provided data on the flow of information from hunters to the final government institution, the recording of wild boars intended for private consumption relies on diverse national systems combining hunting records, veterinary controls, and official databases. In Austria, hunting bag data are collected at district level, and in some provinces, hunters are required to declare whether carcasses are intended for private consumption or to be placed on the market; the estimated number of wild boars intended for private consumption was derived by subtracting carcasses examined by trained persons from total hunting bags. In Belgium, the figures were obtained from government data as the number of hunted wild boars minus those sent to game-handling establishments. In Bulgaria, data were extracted from the electronic “Hunt” module of the Bulgarian Food Safety Agency, an open-access mobile application used by hunters to register hunted animals, collect samples, and record laboratory results, enabling centralized, real-time reporting. In Croatia, wild boar harvest and inspection data are recorded through mandatory paper documentation and the national Central Hunting Register (SLE), with traceability supported by hunting permits, certificates of game origin, and veterinary inspection records. In Poland, all wild boars intended for consumption, including those intended for private use, are subject to mandatory *Trichinella* spp. testing, with samples registered at public or designated laboratories and data consolidated through the Veterinary Inspection system at district, regional, and national levels. In Romania, hunters report carcasses through paper forms to authorized private veterinarians and game management authorities; negative results are transmitted from private veterinarians to county and national sanitary-veterinary authorities, whereas positive results trigger official confirmation testing and further reporting. Overall, the identification of wild boars for private consumption was reported to be based on either direct declarations, mandatory laboratory testing, or estimations derived from hunting and inspection data, depending on the national reporting framework.

#### 3.1.2 Tested animals

In 11 out of the 26 participating countries (42.3%; Bosnia and Herzegovina, Bulgaria, Croatia, Czech Republic, Germany, Lithuania, North Macedonia, Poland, Romania, Serbia and Spain), wild boar meat used for private consumption is subject to mandatory testing for *Trichinella* spp. In addition, in Portugal, testing for *Trichinella* spp. is mandatory only when wild boars are hunted during a drive hunt in designated epidemiological risk areas for *Trichinella* infection, whereas in Italy testing is mandatory only in some regions. In Albania, however, hunting wild game animals is prohibited, and in Turkey, the consumption of wild boar meat is not allowed. For the remaining 11 countries (42.3%; Austria, Belgium, Finland, France, Greece, Latvia, Netherlands, Norway, Slovakia, Sweden, and the United Kingdom), wild boar meat used for private consumption does not legally require testing for *Trichinella* spp. The absence of mandatory *Trichinella* spp. testing policies for privately consumed wild boar meat in 42.3% of the surveyed countries represents a potential risk gap for consumers, particularly in high-prevalence settings.

Of the 11 countries that mandatorily conduct *Trichinella* spp. testing of wild boar meat intended for private consumption, only five countries (Bulgaria, Croatia, North Macedonia, Poland and Romania) provided data on the number of tested wild boars. According to the data provided by Bulgaria and Poland, all wild boars used for private consumption were tested in these countries. However, there are discrepancies between the data provided by these countries within this study and the data that these countries officially report to EFSA. It should also be noted that in Croatia the number of animals reported for private consumption is close to the total number of animals tested according to EFSA, but also much higher than the number of animals tested for private consumption, since testing is mandatory in this country (EFSA and ECDC (European Food Safety Authority and European Centre for Disease Prevention and Control), 2023, EFSA and ECDC (European Food Safety Authority and European Centre for Disease Prevention and Control), 2024, EFSA and ECDC (European Food Safety Authority and European Centre for Disease Prevention and Control), 2025).

There is a marked inconsistency between the number of harvested animals reported by FACE (FACE, 2025) and the number of wild boars tested according to EFSA (EFSA and ECDC (European Food Safety Authority and European Centre for Disease Prevention and Control), 2023, EFSA and ECDC (European Food Safety Authority and European Centre for Disease Prevention and Control), 2024, EFSA and ECDC (European Food Safety Authority and European Centre for Disease Prevention and Control), 2025). In Bulgaria, Germany, Spain and Romania the annual number of hunted wild boars clearly exceeds the number of animals tested for *Trichinella* spp., suggesting that a relevant proportion of carcasses may not be tested despite existing legal obligations. These discrepancies between harvest and testing figures may create a potential risk to consumers particularly in countries where the prevalence of *Trichinella* spp. infection in wildlife is high. Conversely, in Poland, the number of wild boars tested and reported to EFSA is higher than the number of animals harvested according to FACE; this may reflect differences in reporting systems or the inclusion of additional categories of animals in the surveillance data. In Croatia and the Czech Republic, the figures for hunted and tested wild boars are very similar, supporting the reliability of these national datasets.

#### 3.1.3 Trichinella spp. prevalence

Only six countries reported *Trichinella* spp. prevalence in wild boars tested for private consumption, with values ranging from 0.0% in Slovakia to 6.1% in Bulgaria. Of these, five countries have mandatory *Trichinella* spp. testing in place (Table 1). This heterogeneity reflects substantial differences in surveillance objectives, methodologies, and reporting practices across Europe, resulting in a fragmented dataset that complicates direct comparisons. Although most European countries collect some information on wild boar hunting and *Trichinella* spp. prevalence, significant gaps remain, particularly regarding animals intended for private consumption.

Considering the high number of wild boars consumed outside official market channels, systematic testing is crucial to safeguard public health and to prevent human trichinellosis. Harmonized surveillance approaches would provide a more accurate and comparable representation of the epidemiological situation at national level. Furthermore, effective communication and coordination among EU Member States are essential to establish rapid alert mechanisms, given that wildlife migration transcends national borders and globalization increases the risk of pathogen spread (Pozio et al., 2010).

### 3.2 Legal requirements for testing Trichinella spp. in wild boar meat intended for private consumption

In 13 out of 26 surveyed countries, where testing for *Trichinella* spp. in wild boar meat intended for private consumption is mandatory, either nationwide or under specific conditions, more detailed questions were asked regarding the samples (Table 4) and the methods used. The specific muscles used for testing may vary by country (see Table 4 for details).

The diaphragm is the preferred muscle tested for *Trichinella* spp. detection across all 13 countries, in accordance with the regulation (EU) 2015/1375 for wild boar meat intended for the market. According to this regulation, the tongue and the foreleg muscles can be tested instead of the diaphragm; these muscles are tested in eight countries to detect *Trichinella* sp. larvae in wild boar meat intended for private consumption. Intercostal and masseter muscles of wild boars are tested for meat intended for private consumption in four countries even if these muscles are not reported in the regulation. The sensitivity of *Trichinella* spp. larva detection methods can be significantly influenced by the choice of sampled muscle, affecting the ability to compare results across Europe (Gottstein et al., 2009).

#### 3.2.1 Handling test samples

All 13 countries where *Trichinella* spp. testing of wild boar meat intended for private consumption is mandatory provided data on responsibility for sample collection. In all countries, at least one hunting participant is responsible for collecting samples. In nine countries, responsibility is shared among hunters, veterinarians and/or hunting management entities. Hunters are involved in sample collection in 12 countries and are required to undergo training in eight of them. Veterinarians and hunting management entities are involved in seven and four countries, respectively. In Germany, only authorized veterinarians and trained hunters are permitted to collect samples.

Individuals responsible for sample collection are required to deliver samples to the laboratory. In North Macedonia, veterinary ambulances or private clinics contracted by the Food and Veterinary Agency are paid to send samples to accredited laboratories. In Germany, if a hunter delivers a wild boar to a game handling establishment, the hunter is no longer responsible for *Trichinella* spp. testing, and any previous examination commissioned by the hunter is considered invalid. For such establishments, it is obligatory to have samples collected by an authorized veterinarian.

One of the critical control points affecting the outcome of *Trichinella* spp. testing is the accurate sampling of the carcasses (Gajadhar et al., 2019). Samplers must have adequate knowledge of wild boar anatomy, as well as of the procedures for collecting, packaging, labelling, storing and transporting the samples. Regulation (EC) No 853/2004 stipulates that if wild game is placed on the market for human consumption, a trained person can be involved (Anonymous, 2004). If wild boar or other meat is intended for personal use, Regulation (EU) No 2015/1375 stipulates that national risk-mitigation measures apply, rather than uniform EU-wide requirements for *Trichinella* testing. As a result, EU Member States may establish specific national rules and procedures for *Trichinella* control in meat intended for private consumption, while EU legislation provides an overarching framework for official controls when meat is placed on the market.

#### 3.2.2 Sample transport and storage requirements

Specific requirements for the transport and storage of wild boar meat samples before *Trichinella* spp. testing were reported by 11 of 13 countries (84.6%), as Bosnia and Herzegovina and Spain reported no such requirements. Specific requirements on sampling, packaging, labelling, accompanying documentation, pre-transport storage, sample transportation, and carcass storage until the *Trichinella* spp. test result is available, are implemented either partially or fully in most of the 11 countries. Special attention is given to traceability (Bulgaria, Croatia, Czech Republic, Germany, Italy, Poland, Portugal, Romania and Serbia), which includes the identification of each individual sample and associated data related to the hunting area. In Bulgaria, Croatia, Germany, Italy, Lithuania, North Macedonia, Poland, Romania and Serbia, muscle samples must be stored refrigerated to prevent decomposition. A ban or recommendation against freezing samples prior to testing applies in Germany, Lithuania and Poland. In some countries, freezing samples is prohibited, and certain countries also set specific deadlines for submitting samples to the laboratory. Freezing can inactivate larvae that are not resistant to freezing and may consequently reduce the sensitivity of the digestion test. Since game samples are typically stored for some time before transport to the laboratory (Gajadhar et al., 2019), these requirements help ensure reliable testing. In Lithuania, the deadline for sample delivery is 24 h after collection, while in Italy, it is five days. Hunters play a key role in sample collection and delivery to the laboratory. Most countries, including Czech Republic, Germany, Italy, Lithuania, Poland, Romania, and Serbia, require that hunters involved in the process undergo formal training. Similarly, most countries, including Bulgaria, Croatia, Czech Republic, Germany, Italy, Lithuania, North Macedonia, Poland, Portugal, Romania, and Serbia, regulate the procedures for sample collection, storage and transport.

#### 3.2.3 Approved diagnostic methods

The method of artificial digestion is the standard method used in all 13 countries where *Trichinella* spp. detection testing for private consumption is mandatory. The trichinoscopy test is still allowed in Croatia and Romania. The PCR method is also allowed in Croatia. Pooled samples can be tested by artificial digestion in all countries but Lithuania, where samples must be tested individually. In most countries, the sample weight complies with Regulation (EU) 2015/1375.

#### 3.2.4 Test performance

The purpose of this section is to summarize test performance, approval rules, training requirements, and related economic burdens. Laboratory testing of wild boar meat intended for private consumption in all countries, except Spain, is performed in official or accredited laboratories or those designated by national competent authorities such as Veterinary Inspection Services (VIS) or national public health institutes (NPHI). Examination may be performed in both private and official laboratories. However, consistency is lacking, as in Germany, Poland and Spain testing is allowed in private laboratories, provided that they are officially approved and meet specific quality standards (or minimum requirements). Different approaches to laboratory requirements are evident. In Poland, the list of laboratories authorized to test wild boar meat for personal use is published online. Laboratories are obligated to have a quality system (QS) confirmed by an accreditation body (according to ISO 17025) or equivalent QS, and to participate in Proficiency Testing (PT) with a positive result (International Organization for Standardization, 2017). However, the meaning of an “equivalent QS” remains unclear, especially when laboratory performance is not verified by an accreditation body.

As reported above, there is considerable variation in sampling practices. In most countries, *Trichinella* spp. testing must be performed by official laboratories designated by a competent authority, regardless of the intended use of the meat. However, Spain is an exception, as it allows private veterinary clinics to carry out these tests under specific conditions, while countries including Bulgaria, Croatia, Czech Republic, Germany, Italy, Lithuania, North Macedonia, Portugal, Romania, and Serbia maintain strict requirements for official laboratory testing to ensure regulatory compliance and public health safety. In these countries, laboratories conducting *Trichinella* spp. testing must meet ISO/IEC 17025 accreditation requirements and be approved according to Regulation (EU) 2015/1375. This includes use of approved detection methods - primarily MSD, participation in PT programs, and demonstration of staff competence (Marucci et al., 2009). Unlike centralized systems in countries such as Italy, where laboratory approval falls under regional authorities, national oversight remains in place for strategic coordination. Regarding training and personnel, it was observed that, generally, only trained and authorized individuals may perform the test. Training usually includes a theoretical part on parasite biology, public health risks, and EU legislation, and a practical part covering sample preparation, digestion, sedimentation, and microscopy. Training is mostly done by NRLs or designated accredited laboratories. It must be emphasized that according to the survey and EFSA guidelines, insufficient training increases the risk of false-negative results, especially at low levels of infection (<1 larva/g) (EFSA BIOHAZ Panel, 2013).

According to the EU regulation, only carcasses of wild boars intended for the market are regulated for *Trichinella* spp. However, a regulatory gap occurs with wild boar meat intended for private consumption. The EU regulation leaves the issue to the competence of Member States. Data from a European survey and scientific literature review indicate that regulatory rigor, test accessibility, training requirements, and financial support mechanisms vary significantly between countries (Alban et al., 2011). In many countries, hunters or private individuals consuming their own game meat are not bound by the same systematic testing requirements. In practice, this means that consumer protection level is much lower, often depending on voluntary testing, local traditions, or hunters' awareness of zoonotic risks.

#### 3.2.5 Testing costs

The cost of *Trichinella* spp. testing of wild boar meat differs significantly by country. In Bulgaria, Italy, North Macedonia, Poland, Portugal and Serbia, testing is partially subsidized or completely free when legally required. In other countries, including Croatia, Czech Republic, Lithuania and Spain, the cost is covered by the hunters. According to the survey, examination costs ranged from 0.5 to 35 EUR per sample, depending on the region and laboratory. The difference of the test cost among countries may seem significant and is linked to the cost of living in the countries. In Germany, prices are regionally regulated and vary between federal states. In Poland, there is an official price list for testing since the costs are covered by the government. Serbia and Italy (depending on the region) offer financial support for *Trichinella* spp. testing of wild boar meat, in Bulgaria transport costs are supported. In Italy and Spain, some regional authorities include testing as part of broader wildlife disease surveillance programs or offer cost-sharing schemes. In the experience of some authors, excessively low fees may encourage the use of cheaper and less sensitive methods such as trichinoscopy instead of MSD, thereby increasing the risk of false-negative results.

#### 3.2.6 Reporting of analysis results

The reporting of *Trichinella* spp. detection results in wild boar meat intended for private consumption varies among European countries that perform such analyses, with common practices as well as notable differences in reporting structures. According to the questionnaires, the test results are mandatorily reported in 12 of 13 countries since the obligation is not clear for Spain. The analysis results are reported to competent authorities such as national, regional or county-level veterinary or food safety authorities, ministries or national agencies in all the countries, according to the country administrative structure. Mandatory reporting to NRLs as key players for further analysis or risk assessment is in place in Bulgaria, Germany and Italy. The importance of both administrative oversight and scientific analysis is highlighted by the reporting system including competent authorities and other relevant entities (e.g., regional veterinary officers, NRLs).

#### 3.2.7 Mandatory measures and procedures for Trichinella-positive samples

Across the 13 countries where *Trichinella* spp. testing is mandatory for wild boar meat intended for private consumption, the overall reported steps in case of positive samples include: i. the laboratory reporting to relevant authorities; ii. alerting hunters who hunted the *Trichinella*-positive wild boar through traceability records; iii. confirming the presence of the parasite through secondary testing; and iv. ensuring proper disposal of infected carcasses. A significant number of countries, including Bosnia and Herzegovina, Bulgaria and Poland require measures to trace the infected meat by tracking the hunting area, the hunter owner and the wild boar carcass. This is crucial for understanding the origin of infected meat and preventing outbreaks.

The most common standard practice in the participating countries requires positive results to be reported by the respective laboratory to the competent veterinary or food safety authorities. In the Italian region of Campania, official veterinarians or the game owners are responsible for managing positive results, indicating a more localized approach rather than reporting to the national authorities compared to the national reporting systems in other countries. In Poland, hunters report immediately to the regional authorities, and in Portugal, to the national authorities, with proper sample handling also foreseen. In Bulgaria, Croatia, Czech Republic, Germany, Lithuania, Portugal and Romania, the competent authority and the testing laboratory alert the hunter—or the contact person of the hunting team—about the positive test result of the wild boar carcass, to prevent the private consumption of infected meat.

Another widely adopted measure to prevent further spread of infected muscles is the proper disposal and complete destruction of infected wild boar carcasses by competent authorities as animal by-products (ABP). Concerning the categorization of the infected carcass there are differences among the countries (e.g., ABP Category 1 in Croatia, Poland, Portugal and Romania) (Regulation (EC) No 1069/2009). In Poland, the national legislation includes a formalized intervention procedure (Chief Veterinary Officer's instruction) that ensures standardized actions for both wild and farmed game. In Lithuania, the entire carcass and viscera should be incinerated in a certified disposal site at the hunter's expense accompanied by a certificate of destruction or a transfer certificate, meat even if heat-treated should not be consumed, and all tools and the environment where the meat was stored must be properly disinfected. In Germany, while proper disposal is expected, no specific regulations for restriction zones or hunting zones are foreseen as in the case of enzootic diseases like the ASF. In Romania, local hunting associations must implement measures in affected areas, while in Croatia where restriction zones are foreseen. In the three latter countries, hunters play an active role in the proper handling of infected meat and the application of preventive measures. In addition, further testing for confirmation of *Trichinella* spp. presence/identification at the species level is required in several countries by the respective NRLs.

#### 3.2.8 Penalties for non-compliance with Trichinella testing regulations

The questionnaire included two items addressing the penalties applied for non-compliance with *Trichinella* spp. testing regulations for meat intended for private consumption. In Czech Republic, Italy, North Macedonia, Poland, Romania, and Serbia, the confiscation of the meat is a penalty measure, regardless of whether the carcass is negative or positive. Fines are applied in the case of positive samples in Bulgaria, Czech Republic, Germany, Poland, Portugal, and Serbia. Both confiscation and fines are applied in Czech Republic, North Macedonia, Poland, and Serbia. No legal measures are in place in Bosnia and Herzegovina, Croatia, Lithuania and Spain. Additional penalties are implemented in some countries. In Germany, the license for collecting samples may be revoked following repeated non-compliance with sampling procedures or documentation requirements. In Poland, in addition to confiscation and fines, criminal liability may be initiated under the penal code in cases of serious violations, such as placing meat on the market that endangers human or animal health. Furthermore, affected consumers or other parties may seek compensation for damages caused by the illegal placement of meat on the market. In Serbia, legal proceedings may be conducted for cases involving deliberate placement of uninspected meat on the market or professional errors during meat inspection (Table 5). While confiscation and fines are the most common sanctions, the absence of obligatory testing for *Trichinella* spp. of wild boar carcasses intended for private consumption in some countries and the lack of awareness of legal provisions in others may reduce the effectiveness of control programs. In contrast, countries such as Germany, Poland, and Serbia have implemented additional punitive mechanisms, such as criminal prosecution or imprisonment, that may serve as stronger deterrents, although their practical enforcement and impact remain unknown. The European Union mandates systematic testing for *Trichinella* spp. in meat from susceptible species, including wild boar, through Regulation (EU) No 2015/1375. However, exemptions for hunters supplying small quantities directly to final consumers, as applied in the UK, may limit the effectiveness of these safeguards (Food Standards Agency and Animal and Plant Health Agency, 2018).

### 3.3 Awareness-raising communication

The questionnaire included two questions regarding awareness communication on *Trichinella* spp. control, specifically relating to the presence of specific awareness communication in the past two years (2023, 2024) for hunters or the general population. No communication occurred in this period in Albania, Croatia, Greece, Latvia, Lithuania, North Macedonia, Netherlands, Portugal, Serbia and Turkey, while no information was available for Germany. Some form of awareness communication occurred in the past two years in Austria, Belgium, Bosnia and Herzegovina, Bulgaria, Czech Republic, Finland, France, Italy, Norway, Poland, Romania, Slovakia, Spain, Sweden and the United Kingdom. For example, the standard training given to hunters during licensing was considered awareness communication in Austria, France, Italy, Poland and Slovakia. Hunter training in most European countries includes information on *Trichinella* spp. and its control, yet this is aimed only at new hunters and is not regarded as awareness communication in general. Austria and Poland, however, also have additional awareness campaigns running alongside hunter training. Sweden and the United Kingdom have information documents available online, but these are not disseminated at specific times to defined audiences. Interested parties are directed to these resources, if needed. Again, this is not considered general awareness communication, and many European countries make similar information available online, whether through the Food Safety Authority or hunters' associations. In Belgium, information about *Trichinella* spp. occasionally appears as a short news item or in the hunters' association magazine. In some countries, including Bosnia and Herzegovina, Bulgaria, Czech Republic and Romania, general awareness communication included news articles in mainstream news regarding *Trichinella* spp. in wild boar and the risk for human health (following a local outbreak or as a periodical reminder before the hunting season), meetings with hunters to discuss sampling methods and risks, scientific conferences and information disseminated by the Food Safety Authority or the hunters' associations to the general public or the hunter community.

Despite considerable effort in some countries, awareness communication is lacking in other countries. Some countries have a very low prevalence of *Trichinella* spp. in wild boar or other game species, and few or no human outbreaks, making such communication a lower priority. Nevertheless, the risk of an outbreak is not zero, as *Trichinella* spp. is occasionally detected in wild boar. A French case–control study on human trichinellosis showed that patients, even hunters, were unaware of the risks of consuming raw wild boar meat (Peju et al., 2023).

According to the questionnaire, there is no relationship between awareness communication and the prevalence of *Trichinella* spp. in wild boar. Austria occasionally implements campaigns even when prevalence is 0%, whereas other countries with higher prevalence do not.

Moreover, awareness communication should also target populations in countries where traditional, home-made, uncooked or insufficiently cooked pork and wild boar products are part of the diet, as these remain high-risk foods responsible for many trichinellosis outbreaks (Buncic, 2012; Dupouy-Camet and Murrell, 2007). The lack of knowledge in the general population potentially leads to difficulties in diagnosing trichinellosis, as general practitioners are often unaware of the parasite when cases are sporadic (Dupouy-Camet et al., 2002; Dupouy-Camet and Murrell, 2007).

Awareness communication is a proven tool for *Trichinella* spp. control. In Serbia, where home-made pork and wild boar products are common, prevalence in animals and trichinellosis cases have clearly declined over the past decade, partly due to effective communication between medical professionals, public health instances and veterinary authorities (Vasilev et al., 2023).

### 3.4 Main challenges in ensuring Trichinella-free wild boar meat

In Albania, Bosnia and Herzegovina, Croatia, Italy, Lithuania, Poland, Romania, Serbia, and Slovakia, the most frequently reported challenge was hunters not submitting meat for examination. A lack of awareness among hunters and consumers was reported by Bosnia and Herzegovina, Bulgaria, Croatia, Lithuania, and Poland. Lack of free training for hunters was also cited by Croatia, Italy, Lithuania, North Macedonia, and Romania. Insufficient testing facilities or lack of resources were reported by Italy, Lithuania, and Portugal. Bosnia and Herzegovina, Croatia, Italy, Lithuania, and Poland provided multiple challenges, while Austria, Belgium, Czech Republic, Finland, France, Greece, Latvia, the Netherlands, Norway, Sweden and Turkey did not provide any answer (Table 6). These findings indicate that compliance with testing requirements is hindered not only by regulatory gaps but also by behavioral, educational, and logistical barriers. Other reasons for the failure to send muscle samples for *Trichinella* spp. testing are: i. A percentage of wild boars hunted are poached and therefore the samples cannot be sent to official laboratories; and ii. Hunters send muscle samples to the laboratory from only a percentage of the wild boars killed during a hunt. Hunters' failure to submit meat for examination may be linked to low-risk perception, lack of awareness of disease risks, or the inconvenience of testing procedures—factors also identified in other epidemiological studies (Pozio and Murrell, 2006). Insufficient training opportunities and the absence of adequate testing facilities may further reduce compliance, particularly in rural or resource-limited areas (Kirjušina et al., 2015) (Sgroi et al., 2023).

### 3.5 Recommendations for improving control of Trichinella in wild boar meat

When asked about potential measures to improve *Trichinella* spp. control, representatives from Poland, Portugal, Romania, Serbia, Slovakia, and Spain suggested the following measures: strengthening education and training initiatives for hunters and consumers; providing free testing of wild boar samples; raising awareness among hunters and consumers; developing joint cooperation between competent authorities and hunter associations to support prevention, control, and possible eradication of trichinellosis; establishing a clear traceability system for wild boar meat; improving reporting of positive samples and human trichinellosis cases; applying sanctions to hunters and veterinarians who do not comply with regulations or encouraging and promoting research studies aimed at controlling infection on game farms. These recommendations align with current expert consensus that integrated control strategies—combining regulatory enforcement with public education, stakeholder engagement, and targeted research—are the most effective approach to reducing the public health burden of *Trichinella* (Gamble et al., 2000). Enhanced cooperation between authorities and hunting organizations, alongside free testing, could facilitate compliance, while clear traceability systems and improved reporting would strengthen surveillance. Consistent application of sanctions may deter non-compliance, and targeted research could refine preventive strategies, particularly for game farm settings (EFSA BIOHAZ Panel, 2013) (Moral et al., 2022). In summary, the country-level variation in enforcement, awareness, and infrastructure underscores the need for a harmonized, multi-faceted approach to *Trichinella* spp. control in Europe. Aligning regulatory frameworks with education, infrastructure investment, and enforcement could substantially enhance public health protection and mitigate the risk of trichinellosis associated with wild boar meat for private consumption.

### 3.6 Limitations of study

This study is based on expert survey responses and routinely reported European datasets, which are subject to important constraints in scope and completeness. Training structures for meat inspection and *Trichinella* spp. testing were not systematically captured, as the administered questionnaire focused on baseline testing practices rather than on who provides training and how it is implemented, which may limit the interpretation of test performance and capacity across countries. In addition, for some questions on main challenges (e.g. Table 6), several countries provided no answer, including nations with substantial hunting activity; it remains unclear whether these missing responses reflect a genuinely low perceived relevance of *Trichinella* spp. in wildlife or, rather, a lack of centralized information on private game meat distribution and control. The frequent use of “n.a.” (not available) in Table 1, Table 3 also illustrates heterogeneous data availability: in some cases, information is not collected at all within national reporting systems, while in others it was simply not accessible to, or known by, the expert completing the survey. These limitations reduce the precision of cross-country comparisons and underline that the true extent of *Trichinella* spp. exposure through privately consumed wild boar meat is still likely underestimated.

**Table 3:** *Trichinella* spp. surveillance data in wild boar meat across 26 European countries in the 2022–2024 period according to FACE (2025) and EFSA and ECDC (European Food Safety Authority and European Centre for Disease Prevention and Control), 2023, EFSA and ECDC (European Food Safety Authority and European Centre for Disease Prevention and Control), 2024, EFSA and ECDC (European Food Safety Authority and European Centre for Disease Prevention and Control), 2025.

Country^1^	Year^2^	No. of hunted wild boar 3	No. positive/ tested (%) for *Trichinella* spp. 4	Mandatory test
Austria	2022	n.a.^5^	0/38,938	No
	2023	47,821^6^	n.a.^5^	

Belgium	2022	n.a.^5^	0/17,440	No
	2024	3390^7^	n.a.^5^	

Bosnia & Herzegovina	2023	3757	n.a.^5^	Yes

Bulgaria	2022	n.a.^5^	132/7617 (1.7)	Yes
	2023	n.a.^5^	128/31,298 (0.41)	
	2024	16,743	137/8515 (1.6)	

Croatia	2022	n.a.^5^	62/994 (6.2)	Yes
	2023	48,051	65/49,985 (0.13)	
	2024	n.a.^5^	69/45,502 (0.15)	

Czech Republic	2022	n.a.^5^	3/194,769 (0)	Yes
	2023	258,253	1/255,657 (< 0.01)	
	2024	n.a.^5^	n.a.^5^	

Finland	2022	n.a.^5^	1/1044 (0.1)	No
	2023	985	1/595 (0.17)	
	2024	n.a.^5^	1/962 (0.1)	

France	2022	n.a.^5^	1/41,663 (<0.01)	No
	2023	863,124^6^	4/55,035 (<0.01)	
	2024	n.a.^5^	2/46,062 (0.004)	

Germany	2022	n.a.^5^	28/418,567 (0.01)	Yes
	2023	550,551^6^	20/416,930 (<0.01)	
	2024	n.a.^5^	6/464,134 (<0.01)	

Italy	2022	n.a.^5^	7/177,744 (<0.01)	Yes^8^
	2023	n.a.^5^	15/213,445 (<0.01)	
	2024	n.a.^5^	20/227,027 (0.01)	

Latvia	2022	n.a.^5^	37/10,053 (0.37)	No
	2023	30,200^6^	19/8768 (0.22)	
	2024	n.a.^5^	10/6899 (0.14)	

Lithuania	2023	23,100^6^	n.a.^4^	Yes

The Netherlands	2022	n.a.^5^	0/2718	No
	2024	4411	n.a.^4^	

North Macedonia	2022	n.a.^5^	42/2570 (1.6)	Yes
	2023	n.a.^5^	17/4850 (0.35)	
	2024	n.a.^5^	11/3199 (0.34)	

Norway	2022	n.a.^5^	0/219	No

Poland	2022	n.a.^5^	188/213,525 (0.09)	Yes
	2023	173,661^6^	242/219,218 (0.11)	
	2024	n.a.^5^	274/233,246 (0.12)	

Portugal	2022	n.a.^5^	0/622	Yes^8^

Romania	2022	n.a.^5^	43/7029 (0.61)	Yes
	2023	13,690^6^	29/7245 (0.4)	
	2024	n.a.^5^	36/6611 (0.54)	

Serbia	2023	11,220	n.a.^4^	Yes

Slovakia	2022	52,163	0/9789	No
	2023	n.a.^5^	2/9764 (0.02)	

Spain	2022	450,150	643/212,873 (0.3)	Yes
	2023	n.a.^5^	556/210,873 (0.26)	
	2024	n.a.^5^	680/182,549 (0.37)	

Sweden	2022	n.a.^5^	6/113,803 (<0.01)	No
	2023	105,010	3/98,365 (<0.01)	
	2024	n.a.^5^	3/125,022 (<0.01)	

1 Greece and the United Kingdom did not provide any data for the 2022–2024 period. The test for *Trichinella* spp. in wild boars is not mandatory in these two countries.

2 Only years with available data are reported.

3 Data from FACE Report 2025 (FACE, 2025).

4 EFSA Reports (EFSA and ECDC (European Food Safety Authority and European Centre for Disease Prevention and Control), 2023, EFSA and ECDC (European Food Safety Authority and European Centre for Disease Prevention and Control), 2024, EFSA and ECDC (European Food Safety Authority and European Centre for Disease Prevention and Control), 2025).

5 n.a. = data not available.

6 2,023/2024 harvest data. The table reports wild boar harvest numbers from hunting seasons that vary by national regulations, with most data reflecting the 2023 season.

7 Only wild boars hunted in Flanders.

8 In some regions.

**Table 4:** Muscles used for *Trichinella* spp. testing in countries where the analysis of wild boar meat for private consumption is mandatory.

Country	Tested muscles				
	**Diaphragm**	**Tongue**	**Foreleg**	**Intercostal**	**Masseter**
Poland, Romania	✔	✔	✔	✔	✔
Bosnia and Herzegovina, Czech Republic Germany, Italy, Serbia, Spain	✔	✔	✔	n.t.^1^	n.t.^1^
Portugal	✔	✔	n.t.^1^	✔	✔
Lithuania	✔	n.t.^1^	n.t.^1^	✔	✔
Croatia	✔	n.t.^1^	✔	n.t.^1^	n.t.^1^
Bulgaria, North Macedonia	✔	n.t.^1^	n.t.^1^	n.t.^1^	n.t.^1^

1 n.t. = not tested.

**Table 5:** Reported penalties for non-compliance with *Trichinella* testing regulations for meat intended for private consumption.

Penalty type	Countries
Confiscation of the meat	Czech Republic, Italy, North Macedonia, Poland, Romania, Serbia
Fines for positive samples	Bulgaria, Czech Republic, Germany, Poland, Portugal, Serbia
Both confiscation and fines	Czech Republic, North Macedonia, Poland, Serbia
No legal measures	Bosnia and Herzegovina, Lithuania, Spain

**Table 6:** Main challenges reported in ensuring *Trichinella*-free wild boar meat for private consumption.

Challenge	Countries
Hunters not submitting meat for examination	Albania, Bosnia and Herzegovina, Croatia, Italy, Lithuania, Poland, Romania, Serbia, Slovakia
Lack of awareness among hunters and consumers	Bosnia and Herzegovina, Bulgaria, Croatia, Lithuania, Poland
Lack of free training for hunters	Croatia, Italy, Lithuania, North Macedonia, Romania
Insufficient testing facilities or a lack of resources	Italy, Lithuania, Portugal
Multiple challenges reported	Bosnia and Herzegovina, Croatia, Italy, Lithuania, Poland
No answer provided	Austria, Belgium, Czech Republic, Finland, France, Greece, Latvia, Netherlands, Norway, Sweden, Turkey

## 4 Conclusions

This study provides the first European-level overview of *Trichinella* spp. detection in wild boar meat intended for private consumption, showing that this meat represents a substantial yet poorly quantified component of human exposure in Europe. It reveals marked inconsistencies between hunting bag statistics and *Trichinella* spp. testing data, particularly in countries with legal requirements for testing self-consumed meat, indicating that a proportion of wild boar carcasses may bypass inspection and enter private consumption channels. These gaps and inconsistencies highlight that current surveillance and reporting mechanisms are insufficient to generate a coherent, comparable picture of the risks associated with privately consumed wild boar meat, and that more effective systems are needed to capture and link data on hunting bags, testing coverage, private consumption and human trichinellosis cases. A multidisciplinary One Health approach, combining hunter and consumer education, accessible and reliable meat inspection, and effective regulatory frameworks, is essential to reduce the risk of *Trichinella* infection associated with game meat. In this context, ongoing ASF control efforts—based on systematic carcass detection and removal and wild boar population management—may offer opportunities to strengthen *Trichinella* spp. surveillance in wild boar populations and improve detection of infected animals. By drawing attention to key knowledge gaps and weaknesses in current data flows, this work aims to support the development of safer handling and consumption practices and to contribute to enhanced protection of public health in Europe.

## Declaration of generative AI in scientific writing

The authors declare that no Generative AI was used in the creation of this manuscript.

## CRediT authorship contribution statement

**Mariana Rodrigues:** Writing – review & editing, Writing – original draft, Methodology, Investigation, Formal analysis, Data curation, Conceptualization. **Edoardo Pozio:** Writing – review & editing, Methodology, Investigation, Formal analysis, Data curation. **Vincenzo Veneziano:** Writing – review & editing, Methodology, Investigation, Formal analysis, Data curation. **Famke Jansen:** Writing – review & editing, Methodology, Investigation, Formal analysis. **Thomai Lazou:** Writing – review & editing, Methodology, Investigation, Formal analysis. **Sorin Daniel Dan:** Writing – review & editing, Methodology, Investigation, Formal analysis. **Miroslaw Różycki:** Writing – review & editing, Methodology, Investigation, Formal analysis. **Jelena Petrović:** Writing – review & editing, Methodology, Investigation, Formal analysis. **Madalena Vieira-Pinto:** Writing – review & editing, Writing – original draft, Methodology, Investigation, Formal analysis, Data curation.

## Ethics approval and consent to participate

The current study was approved by the Ethics Committee for Biomedical Activities of the University of Naples Federico II through Decision PG/2025/0133225. All participants signed a specific informed consent form before completing the questionnaire.

## Funding

This publication is based upon work from COST Action Safety in the Game Meat Chain (SafeGameMeat, CA22166), supported by 10.13039/501100000921COST (10.13039/501100000921European Cooperation in Science and Technology).

## Declaration of competing interest

The authors declare that they have no known competing financial interests or personal relationships that could have appeared to influence the work reported in this paper.

## Data Availability

All relevant data are within the paper.

## Appendix A Supplementary data

Survey: *Trichinella* testing on Wild Boar Meat used for Private Consumption
