# Supplementary material for: Trichinella detection in wild boar meat intended for private consumption: Insights from a European study
Source: Food Waterborne Parasitol. 2026 Sep 3;44:e00358. doi: 10.1016/j.fawpar.2026.e00358 (PMC13590011; doi:10.1016/j.fawpar.2026.e00358)
Supplement: Supplementary file 1 [file mmc1.pdf]

Survey: Trichinella testing on Wild Boar Meat used for Private Consumption

This questionnaire is conducted as part of the activities of the SafeGameMeat COST Action CA22166 (https://safegamemeat.eu) in collaboration with the University of Trás-os-Montes e Alto Douro (UTAD). It was developed within the scope of Working Group 4 (WG4), which focuses on biological hazards in game meat. One such hazard is the parasite *Trichinella* spp. in wild boar meat.

According to COMMISSION IMPLEMENTING REGULATION (EU) 2015/1375, wild boar meat intended for commercial sale (see definition below[1]) must undergo official controls for *Trichinella*. However, this regulation does not apply to meat intended for private consumption.

This questionnaire aims to collect information from various European countries regarding the legal requirements for *Trichinella* control in wild boar meat consumed privately. The data gathered will provide insights into the national and regional regulations across the European Union (EU) that govern testing requirements for privately consumed wild boar meat. Your responses will contribute to a better understanding of the regulatory and practical approaches to safeguarding public health in relation to wild boar meat consumption.

[1] ***Placing on the market*** refers to holding food or feed for sale, including offering it for sale or any other form of transfer, whether free of charge or not, as well as the sale, distribution, and other forms of transfer themselves. Private consumption is not included (REGULATION (EC) No 178/2002).

We kindly ask for your cooperation in completing this questionnaire by **April 30, 2025**.

If you have any questions regarding the questionnaire, please contact **Prof. Madalena Vieira-Pinto** at mmvpinto@utad.pt.

We assure the confidentiality of individual responses. Please read and complete the informed consent form above.

Thank you for your time and collaboration.

1. **INFORMED CONSENT FORM**

   PARTICIPATION IN SCIENTIFIC RESEARCH

   Survey: Trichinella testing on Wild Boar Meat used for Private Consumption

   Please read the following information carefully. If you identify any inaccuracies or lack of clarity, please feel free to request additional clarification. If you agree with the proposal presented, please sign this document.

   Dear Participant,

   We invite you to participate in a research study conducted by the University of Trás-os-Montes e Alto Douro (UTAD) in collaboration with COST Action Safe Game Meat. Before you decide, it is important to understand the purpose of the research and what your participation will involve. Please read the following information carefully. Feel free to ask any questions before agreeing to take part (mmvpinto@utad.pt).

   The study aims to collect information across European Union (EU) member states regarding national and regional legal **requirements** on *Trichinella* testing of wild boar used for private consumption and testing requirements for. Your responses will help improve the understanding and development of public health policies. Your participation involves completing a questionnaire about the regulations and practices concerning **Trichinella testing on Wild Boar Meat used for Private Consumption** in your country.

   The questionnaire will be answered by one entity from each EU country, identified as having the most knowledge on this topic. The study will require only one response and no follow-up sessions. Participation is entirely voluntary. You are free to choose not to participate or withdraw at any time without providing a reason and without any penalty.

   All information provided will remain anonymous and confidential. Your answers will be used exclusively for statistical and research purposes. No personal identifying information will be disclosed.

   There are no known risks associated with your participation. While you will not receive any financial or other compensation, your contribution will help improve knowledge and policies related to wild boar meat safety. If you have any questions, require further information, or wish to withdraw from the study, you may contact the lead researcher: Madalena Vieira-Pinto (mmvpinto@utad.pt)

   Participant's consent:

   I declare that I have understood the objectives of what was proposed and explained to me by the researcher, that I have been given the opportunity to ask all the questions about this study and for all of them I have obtained an enlightening answer, so I agree to participate in it. *

   ◯ Yes

   ◯ No

2. Country *

   ____

3. Profession (Ex: Academic Researcher, Hunter, Representative of the Hunting Association, Competent Authority, Representative of the Reference Laboratory, Other...) *

4. Affiliation *

5. Email *

6. Is there any data (between 2020-2024) on the number of wild boars used for **private consumption** in your country? *

- ◯ Yes (It is also valid to answer Yes if you know the figures at regional level)
- ◯ No
- ◯ I don´t know

7. Please specify the data on the number of wild boars used for **private consumption** in your country. *

Please describe number and year (ex: 2000 wild boars/2022)

8. Is there any data on the prevalence of Trichinella infection in wild boars hunted for **private consumption** in your country? *

- ◯ Yes (It is also valid to answer Yes if you know the figures at regional level)
- ◯ No
- ◯ I don´t know

9. Could you kindly provide the numbers. *

Prevalence of Trichinella infection in wild boars hunted for private consumption in your country.
<u>Please describe positives cases and total tested (ex: 20 positive case/2000 wild boars tested)</u>

10. Have there been any specific awareness **communication** in the last two years for hunters or population in your country regarding Trichinella control? *

- ◯ Yes (It is also valid to answer Yes if there is any at regional level)
- ◯ No
- ◯ I don´t know

11. Please, describe the specific awareness **communication** in the last two years for hunters or population in your country regarding Trichinella control. *

Specify the target audience and the **type** of awareness.

12. In your country, does wild boar meat used for self-consumption have to be legally analyzed for Trichinella? *

- ◯ Yes, in all cases
- ◯ In some cases
- ◯ Another situation
- ◯ No, never
- ◯ I don´t know

13. Please describe the "Some cases" or "Another situation" *

14. Mention the name of the legislation that regulates this matter. *

15. Which part(s) of muscles can be use to test? *

- [ ] Diaphragm
- [ ] Masseter Muscle (Jaw Muscle)
- [ ] Foreleg
- [ ] Intercostal
- [ ] Shoulder Muscles
- [ ] Tongue
- [ ] Other
- [ ] I don´t know

16. **If your response it was "Other"**, please describe the part(s) of muscles can be use to test.

17. Whose obligation to <u>collect</u> samples? *

*Trained Hunter is a hunter that have specific training for the correct procedure to collect the samples.

- [ ] Trained Hunter*
- [ ] Hunter
- [ ] Hunting Management Entity
- [ ] Veterinarian
- [ ] Other
- [ ] I don´t know

18. **If your response was "Other"**, please **decribe** the other identity that have obligation to <u>collect</u> samples.

19. Whose obligation to <u>deliver</u> samples? *

*Trained Hunter is a hunter that have specific training for the correct procedure to collect the samples.

- [ ] Trained Hunter*
- [ ] Hunter
- [ ] Hunting Management Entity
- [ ] Veterinarian
- [ ] Other
- [ ] I don´t know

20. **If your response it was "Other"**, please **decribe** the other identity that have obligation to <u>deliver</u> samples.

21. Are there specific requirements for the transport and storage of wild boar meat before Trichinella testing is completed? *

- ◯ Yes
- ◯ No
- ◯ I don´t know

22. Please, decribe specific requirements **<u>(temperature, time until the testing, traceability, legal requirements or good practices)</u>** for the transport and storage of wild boar meat before Trichinella testing is completed. *

23. Which test method(s) are permitted? *

- [ ] Method of artificial digestion
- [ ] ELISA
- [ ] PCR
- [ ] Trichinoscope
- [ ] Other
- [ ] I don´t know

24. **If your response it was "Other",** describe the method and if it is based on legal regulation.

25. Regarding Method Artificial Digestion, samples **must be** analysed individually or can be used as pool samples? *

- ◯ Must be analysed individually
- ◯ Can be analysed as pool samples
- ◯ I don´t know

26. Regarding Method Artificial Digestion, what is the **minimum amount of muscle** required from each animal for Trichinella testing? *

Based on question " Which part(s) of muscles can be use to test?"
According Reg. UE 2015/1375: minimum weight of 5g for digestion

	According Reg. UE 2015/1375	Other	NA (Non aplicable)
Diaphragm	◯	◯	◯
Masseter Muscle	◯	◯	◯
Intercostal	◯	◯	◯
Shoulder Muscles	◯	◯	◯
Foreleg	◯	◯	◯
Tongue	◯	◯	◯
Other	◯	◯	◯

27. **If your response it was "Other"** please specify what is the minimum amount of muscle (grams) required from each animal for Trichinella testing.

28. **If you have additional comment** relate to question "Regarding Method Artificial Digestion, what is the **minimum amount of muscle** required from each animal for Trichinella testing?", **please add here.**

29. Where can be performed the test? *

- [ ] Reference laboratory
- [ ] Private laboratory
- [ ] Designated laboratory by the Competent Authority
- [ ] Veterinary Clinic
- [ ] Veterinarian
- [ ] Other
- [ ] I don´t know

30. **If your response it was "Other"**, please specify the test.

31. Is there any specific requirement for approval of the laboratories conducting Trichinella testing of wild boar meat for private consumption? If yes, please describe. *

32. Please, describe the test needed to have any specific training or authorization. *

33. Is there any cost associated with this analysis? *

- ( ) Yes
- ( ) Sometimes
- ( ) No
- ( ) I don´t know

34. Please, specify the cost and who needs to pay *

35. Are there any subsidies or financial support available to hunters to cover Trichinella testing costs? *

- ◯ Yes
- ◯ Sometimes
- ◯ No
- ◯ I don´t know

36. Please, specify the subsidies or financial support available to hunters to cover Trichinella testing costs. *

37. Are the results of the analyses mandatorily reported? *

- ◯ Yes
- ◯ No
- ◯ I don´t know

38. Please specify to **whom** the analysis results are reported. *

Example: Competent Authority

39. In case of a positive sample, what measures must be adopted and by whom? *

Example: Report the authorities, Report the hunter, Report the hunting zone, Eliminate properly by products as M1, restrictions....)

40. In case of a positive sample, what are the mandatory steps? *

Was a second test performance for confirmation? Where is doing that test?
What happens to the meat and viscera until the confirmation and after a confirmation?

41. What penalties are in place for non-compliance with Trichinella testing regulations for meat used for private consumption? *

- [ ] None
- [ ] Fines
- [ ] Confiscation of meat
- [ ] Other
- [ ] I don´t know

42. **If you response it was "Other"** please, describe the penalties are in place for non-compliance with Trichinella testing regulations for meat used for private consumption.

43. What challenges does your country face in ensuring Trichinella-free wild boar meat for private consumption?

Examples:
Insufficient testing facilities:
Lack of awareness among hunters and consumers;
Lack of free training for hunters;
Hunters did not bring the meat for examination;
The occurrence of professional errors during the Trichinella examination;
Others.

44. Provide, if you want it, any comment to improve control of Trichinella in wild boar meat for private consumption in your country

Este conteúdo não foi criado nem é aprovado pela Microsoft. Os dados que submeter serão enviados para o proprietário do formulário.

Microsoft Forms
